# Cytoprotective effects of ginsenoside Rd on apoptosis-associated cell death in the isolated human pancreatic islets

**DOI:** 10.17179/excli2019-1698

**Published:** 2019-08-22

**Authors:** Maryam Kaviani, Somayeh Keshtkar, Negar Azarpira, Mahdokht Hossein Aghdaei, Bita Geramizadeh, Mohammad Hossein Karimi, Ramin Yaghobi, Elaheh Esfandiari, Alireza Shamsaeefar, Saman Nikeghbalian, Ismail H. Al-Abdullah

**Affiliations:** 1 Transplant Research Center, Shiraz University of Medical Sciences, Shiraz, Iran; 2 Department of Molecular Medicine, School of Advanced Technologies in Medicine, Tehran University of Medical Sciences, Tehran, Iran; 3 Shiraz Organ Transplant Center, Shiraz University of Medical Sciences, Shiraz, Iran; 4 Department of Translational Research and Cellular Therapeutics, Diabetes and Metabolism Research Institute, Beckman Research Institute of City of Hope, Duarte, USA

**Keywords:** apoptosis, culture, Ginsenoside Rd, human pancreatic islets, insulin, transplantation

## Abstract

Ginsenoside Rd (GS-Rd), one of the main pharmacologically active components of ginseng, has shown the potential to stabilize mitochondrial membrane integrity and decrease apoptotic death in neuronal and non-neuronal cells. The present study aimed to evaluate the effect of this bioactive molecule on the apoptosis-associated cell death in human pancreatic islets. In this regard human pancreatic islets were isolated and grouped for the treatment with GS-Rd. The isolated islets were treated with different concentrations of GS-Rd. After 24 and 72 h of incubation, the islets were evaluated in terms of viability, *BAX*, *BCL2*, and insulin gene expression, BAX, BCL2, and caspase-3 protein expression, apoptosis, and glucose-induced insulin/C-peptide secretion. Our results revealed the islet survival was significantly decreased in the control group after 72 h of incubation. However, GS-Rd inhibited the progress of the islet death in the treated groups. TUNEL staining revealed that the preventive effect of this molecule was caused by the inhibition of apoptosis-associated death. In this regard, the activation of caspase-3 was down-regulated in the presence of GS-Rd. GS-Rd did not exhibit undesirable effects on glucose-induced insulin and C-peptide stimulation secretion. In conclusion, GS-Rd inhibited the progress of death of cultured human pancreatic islets by diminishing the apoptosis of the islet cells.

## Introduction

Islet transplantation has been considered as an effective therapeutic approach in diabetes type 1. However, the islet loss subsequent to pancreatic islets isolation and culture is a substantial challenging aspect of islet transplantation (Kim et al., 2009[9]). This situation leads to a need for multiple donor islet infusions to attain the desired outcome. Apoptosis has been accepted as a main cause of islet loss. Apoptosis occurs through two main pathways, extrinsic and intrinsic. In the intrinsic pathway, the integrity of mitochondrial outer membrane is compromised; this leads to the release of cytochrome *c *and triggers the apoptotic program. It has been reported that the members of *BCL2* family regulate the progress of cell apoptosis. Anti-apoptotic and pro-apoptotic transcriptional genes like *BCL2* and *BAX*, respectively, are involved in this family. *BAX* gene functions as an apoptotic gene which leads to the release of cytochrome *c *and promotes apoptosis. On the other hand, *BCL2* acts as an anti-apoptotic gene and prevents apoptotic-associated death (Korsmeyer, 1999[10]). The interaction between apoptotic and anti-apoptotic proteins determines the survival of the cells. 

Researchers are trying to find appropriate substances to protect the islets in facing apoptosis (Kaviani et al., 2019[6][7]; Keshtkar et al., 2019[8]). Hara et al. reported that green-tea polyphenol(-)-epigallocatechin-3-gallate has protective properties in the isolated pancreatic islets. This agent seems to be involved in the improvement of pancreatic islets survival rate and maintenance of their function (Hara et al., 2007[4]). Toso et al. also studied the effect of Liraglutide, as a peptide like human glucagon, on human islet viability, *in vitro*. They found out the presence of liraglutide results in the reduction of apoptosis and the improvement of insulin secretion in human islets (Toso et al., 2010[19]). Recently, researchers have suppressed palmitate-induced apoptosis in MIN6N8 pancreatic β-cells using ginsenoside Rg3, a major component isolated from ginseng. They reported that this molecule inhibits the progression of type 2 diabetes through the prevention of free fatty acids-mediated β-cells loss (Kim et al., 2009[9]). Ginsenosides are the main active pharmacological ingredients of ginseng. The major ginsenosides are Rb1, Rb2, Rc, Rd, Re, Rf, Rg1, Rh1, and Rh2 that are well isolated and characterized. Previous experimental and clinical studies provided evidence for the application of ginsenosides in multiple diseases.

One of the main active ingredients of ginsenoside is Dammar-24 (25)-ene-3b,12b, 20(S)-triol-(20-O-b-D-glucopyranosyl)-3-O- b-D-glucopyranosyl-(1→2)-b-D-glucopyranoside (ginsenoside Rd: GS-Rd) (Nah et al., 2007[17]; Yang et al., 2007[20]). Investigation of the effect of GS-Rd on neuronal protection in hippocampus showed this agent results in the stabilization of mitochondrial membrane potential and decrease of apoptotic death in them (Ye et al., 2009[22]). The surveys showed that GS-Rd protected the neurons (Zhang et al., 2012[24]) and revealed its efficacy and safety in the treatment of acute ischemic stroke (Liu et al., 2009[15]). On the other hand, GS-Rd also prevents apoptosis in non-neuronal cells. In this regard, Tamura et al. showed the protection feature of this agent against the irradiation-induced apoptosis in the rats' intestinal epithelial cells. GS-Rd reduced the *BAX/BCL2* and *BAX/BCL-xL* ratios, cytochrome *c *release, and caspase-3 activation (Tamura et al., 2008[18]).

According to the importance of the cell mass and function in the islet transplantation outcome, the present study was designed to evaluate the cytoprotective properties of GS-Rd in the isolated human pancreatic islands. We focused on *BCL2* family members and caspase-3 activation as possible mediators of apoptosis in the pancreatic islets.

## Materials and Methods

### Human pancreatic islet isolation and culture

This study is an experimental study. We applied the pancreases from brain-dead heart beating donors with these criteria: age 18 to 70 years; lack of type 1 and 2 diabetes mellitus, severe liver, kidney, and heart diseases; no evidence of microbial infections, less than 12 hours of cold ischemia time; and hospitalization stay ≤ 4 days. The exclusion criterion were the pancreases which failed to meet the inclusion criteria.

Human islet isolation was established according to a standard protocol with some modifications (Azarpira et al., 2014[1]; Iglesias et al., 2012[5]). Human brain-dead donor's pancreas was used in accordance with the ethical standards of the Ethics Committee of Shiraz University of Medical Sciences (document ID: IR.SUMS.REC.1394.S1141) and with the 1964 Helsinki declaration and its later amendments or comparable ethical standards. Ductal distension was done with collagenase and neutral protease (Serva, Germany). After enzymatic and mechanical digestion in Ricordi chamber, COBE 2991 cell processor was recruited to purify the islets on Biocoll (Biochrom, Germany) gradients. The total islet count was determined using dithizone staining, as islet equivalents (IEQ). Islets were cultured in CMRL 1066 (Gibco, UK) supplemented with 1 % FBS (Gibco, UK), 1 % antibiotic (Biosera, France), and 6.25 µg/ml ITS (Sigma, Germany) in 5 % CO_2_ at 37 °C. After overnight culture, the islets were treated with GS-Rd (Sigma, Germany) 0, 0.1, 1, or 10 µM in 5 % CO_2_ at 37 °C for 24 and 72 hours. Next, the islets were tested in triplicate. 

### Live-dead assay

To determine the effect of GS-Rd on the viability of the islets, we applied fluorescent staining method. In this regard, the viability of the islets was determined under fluorescent microscope (CKX53, Olympus, Japan), using 5 mg/ml Fluorescein diacetate (FDA) and 2 mg/ml propidium iodide (PI), both of them from Sigma, Germany. FDA and PI were recruited for the staining of live and dead cells, respectively. The viability rate was estimated by the percentage of live cells in the islets.

### Gene expression analysis

To evaluate the gene expression profile in different groups, RNA extraction was performed following the manufacturer's protocol for the RNA-Sol isolation kit (Alphabio, Canada). After confirmation of RNA integrity using the analysis of the Abs260 nm/Abs280 nm absorption ratio, cDNA was immediately synthesized according to the PrimeScript TM RT Reagent Kit (Takara, Japan). The following primers were designed by NCBI tool Primer BLAST and used in this study: human insulin (F:5´-CTTCTACACACCCAAGACCC-3´; R: 5´-CTGGTACAGCATTGTTCCAC-3´), human caspase-3 (F:5´ ACTCCACAGCACCTGGTTATT-3´; R: 5´-TCTGTTGCCACCTTTCGGTT-3´), human *BAX* (F:5´- TTCTGACGGCAACTTCAACT-3´; R: 5´- GGAGGAAGTCCAATGTCCAG-3´), human *BCL2* (F:5´-GATGGGATCGTTGCCTTATGC-3´; R: 5´-CAGTCTACTTCCTCTGTGATGTTGT-3´), and human Glyceraldehyde 3-phosphate dehydrogenase (*GAPDH*) (F:5´-GCTCATTTCCTGGTATGACAACG-3´; R: 5´-CTCTCTTCCTCTTGTGCTCTTG-3´), as a housekeeping gene. Real time RT-PCR was done according to the SYBR® Premix Ex TaqTM II kit (Takara, Japan), using Applied Biosystems StepOnePlus™ Real-Time PCR System (ABI, USA). The fold changes of the genes were calculated by the 2^-ΔΔCT^. The melt curves were analyzed for each reaction to identify non-specific products.

### Protein expression analysis

After fixation of the islets in 10 % buffered formalin, they underwent embedding in agar, and the second embedding in paraffin was performed. The blocks were sectioned at 5 µm. Next, de-paraffinization was done for immunocytochemistry analysis. The slides were incubated with primary antibodies including anti-human insulin, anti-human BAX, anti-human BCL2, and anti-human caspase-3 (All from Abcam, USA), at 4 °C overnight. After washing, the secondary antibody (Abcam, USA) was used for the detection. The nuclei were stained by hematoxylin. The H-score was applied to estimate the positive level of the islets, as the following formula: H score = 1 × (% light staining) + 2 × (% moderate staining) + 3 × (% strong staining) (Detre et al., 1995[2]).

### Apoptosis detection assay

Terminal deoxynucleotidyl transferase-mediated dUTP nick end labeling (TUNEL) assay was conducted on the sections of paraffin embedded islets using Click-iT® Plus TUNEL Assay (Life Technology, USA). Briefly, TdT reaction was done on de-paraffinization slides for 60 minutes at 37 °C. After washing with 3 % BSA and 0.1 % Triton® X-100 in PBS, Click-iT® Plus reaction was performed for 30 minutes at 37 °C. Then, washing was done with 3 % BSA in PBS. After washing in PBS, nuclei staining was done with DAPI solution. The slides were imaged and analyzed under fluorescence microscope (CKX53, Olympus, Japan). The percentage of the positive cells was calculated by the following formula: Σ (the number of green stained nuclei/the number of blue stained nuclei) ×100.

### Glucose-induced insulin and C-peptide secretion

To elucidate the functionality of the isolated islets, we washed them with PBS. Next, they were incubated for 2 h with RPMI 1640 without glucose (Gibco, Germany) supplemented with 0.5 % BSA and 2.8 or 20 mM glucose. The conditioned media was collected from different wells and stored at -80°C until evaluation. Insulin and C-peptide ELISA kit (Monobind, USA) were used to determine the secreted insulin and C-peptide. The concentrations were evaluated with microplate spectrophotometer and the stimulation index was calculated by dividing the value of insulin/C-peptide secretion in 20 mM glucose by that obtained upon 2.8 mM glucose stimulation (Labriola et al., 2007[11]).

### Statistical analysis

SPSS software (version 24; SPSS Inc) was used for statistical analysis and Graph Pad Prism 6 was applied for drawing the charts. The analysis was carried out with Kruskal-Wallis and post-hoc tests. The data are presented as mean ± SEM, *p*<0.05 was considered significant. 

## Results

### Preservation of the morphology and the significant decrease of the islets death in the presence of GS-Rd

**Figure 1:**
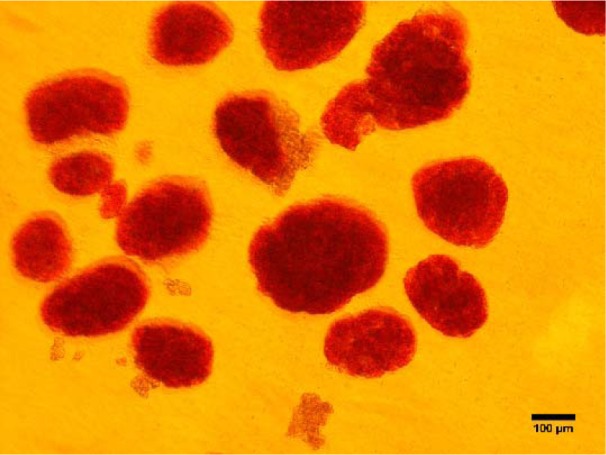
The purified human pancreatic islets. Dithizone stains the islets red.

The human pancreatic islets were isolated, using an enzymatic and mechanical method. After purification process, the purity of the obtained islets was more than 75 %. Dithizone staining confirmed the presence of the islets (Figure 1(Fig. 1)). Evaluation of the morphology revealed the spherical shape of the islets in the presence and absence of GS-Rd. Therefore, GS-Rd did not affect the typical spherical shape of the islets in all studied concentrations.

**Figure 2:**
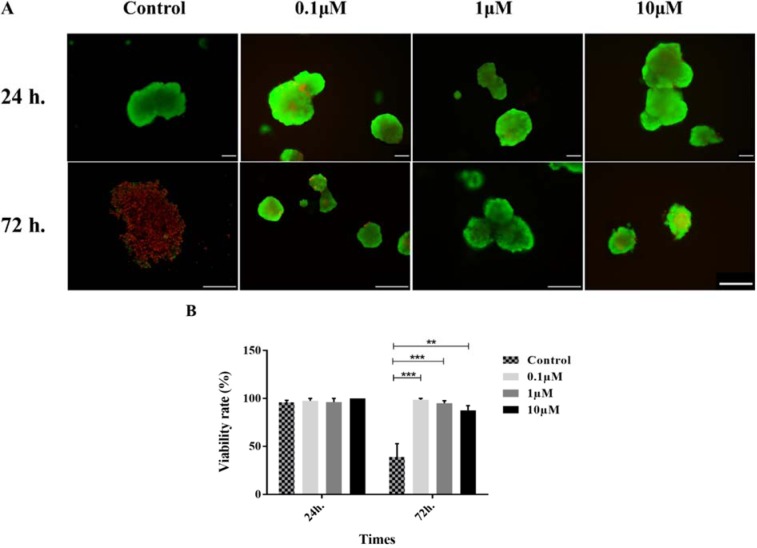
Human pancreatic islets viability. Staining of viable (green) versus dead (red) cells using FDA and PI, respectively (A). The percentage of viable cells in different concentrations of GS-Rd (B). Data are presented as mean ± S.E. **p<0.01, and ***p<0.001. Scale bars = 100 μm for all images

The evaluation of the viability of the islets was performed by fluorescent microscopy. As shown in Figure 2(Fig. 2), no significant difference was found between different groups at 24 h treatment. However, an apparent reduction of the islet survival was detected in the control group at 72 h of incubation. Our findings showed that GS-Rd preserved the survival of the islets in all studied concentrations (*p<*0.001). 

### Fluctuations of the gene's markers associated with apoptosis pathway

**Figure 3:**
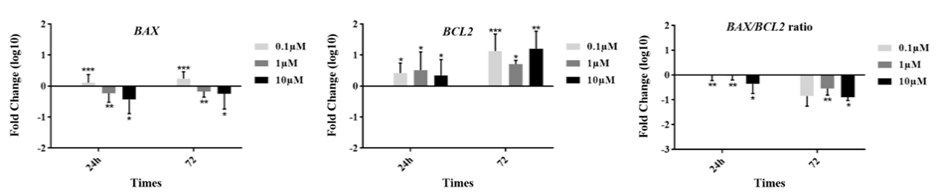
Apoptotic genes expression in different concentrations of ginsenoside Rd. The data show fluctuations in the expression of *BAX* and *BCL2* at the gene level. Ginsenoside Rd showed the potential to decrease the *BAX/BCL2* ratio in different treated groups. ^*^ *p≤0.05*, **p<0.01, and ***p<0.001

To examine the expression of *BCL2* family members including *BAX* and *BCL2* genes in the groups, we analyzed their mRNA levels 24 and 72 h after incubation by real time RT-PCR method (Figure 3(Fig. 3)). In the presence of GS-Rd, the expression of *BAX* was significantly downregulated at 1 and 10 µM concentrations at 24 h (*p=*0.002) and 72 h (*p*=0.003) of incubation. Interestingly, we observed a significant upregulation in a low dose of GS-Rd at two-point times. On the other hand, we detected a considerable upregulation of *BCL2 *in all doses of GS-Rd exposure for 24 h (*p*=0.004) and 72 h (*p*=0.002). According to the importance of the *BAX/BCL2* ratio in the determination of the cell fate, we calculated this ratio. Our findings indicated that *BAX/ BCL2* ratio was significantly decreased in all treated groups at 24 h (*p*=0.006) and 72 h (*p=*0.023). 

### The impact of GS-Rd on the expression of apoptotic proteins

To determine whether apoptotic and anti-apoptotic protein expression patterns were changed after GS-Rd treatment, we performed the quantitative analysis by immunocytochemistry. Our results demonstrated that GS-Rd potentiated the human pancreatic islets to inhibit the intrinsic pathway of apoptosis through the reduction of active caspase-3.

After 24 h of exposure with GS-Rd, the BAX protein was non-significantly reduced in all treated groups. However, the BCL2 protein was non-significantly increased in a dose-dependent manner. The H-score of active caspase-3 was decreased dramatically at the high dose of GS-Rd.

**Figure 4:**
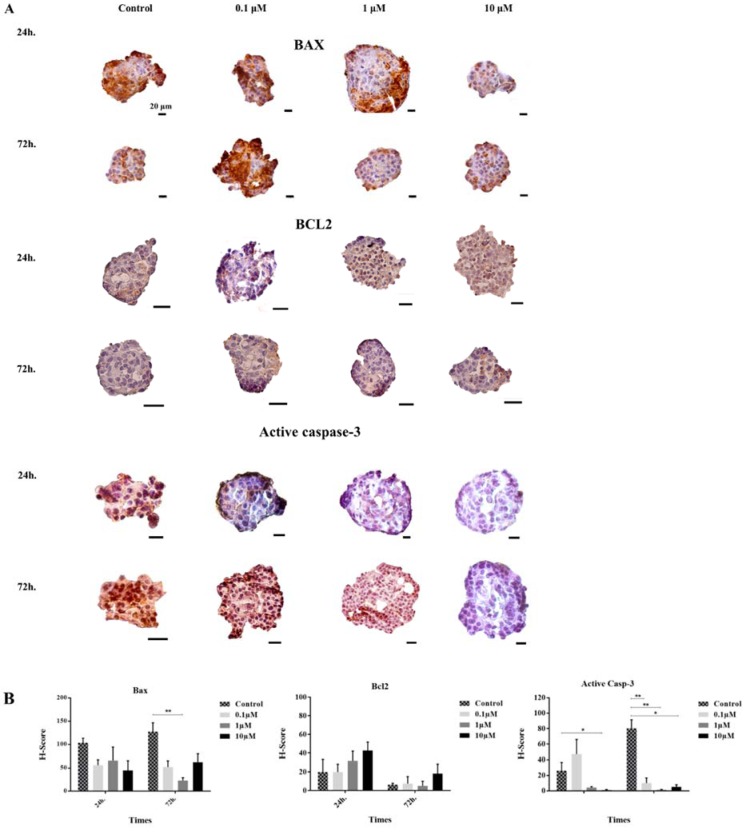
Immunohistochemical analysis of BAX, BCL2, and active caspase-3. The positive areas of antibodies and the nuclei are brown HRP-DAB stained and blue hematoxylin nuclear counterstained, respectively (A). The histograms demonstrate the decrease and increase of apoptosis related proteins in different groups (B). The error bars represent the standard error of the mean. *p<0.05, and **p<0.01

After 72 h of incubation with GS-Rd, the expression of BAX protein was decreased, but this reduction was significant only for 1 µM dose of GS-Rd. The expression of *BCL2* gene was increased non-significantly at 0.1 and 10 µM. Finally, we found out GS-Rd treatment substantially inhibited the activation of active caspase-3 (Figure 4(Fig. 4)).

### A significant reduction in the TUNEL positive islet cells in the presence of GS-Rd

**Figure 5:**
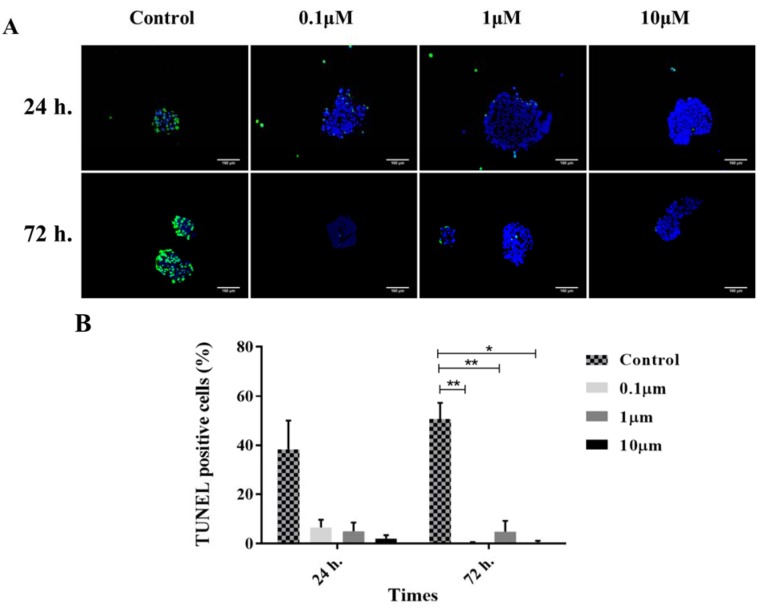
TUNEL assay. The merged images of TUNEL positive cells (green channel) and DAPI nuclear counterstained cells (blue channel) (A). The histograms demonstrate the decrease of TUNEL-positive cells in different treated groups with ginsenoside Rd (B). The error bars represent the standard error of the mean. Scale bars = 160 µm. *p<0.05, and **p<0.01

In this study, TUNEL assay was applied to elucidate the effect of GS-Rd on the apoptosis. In the control groups, we observed a great number of TUNEL positive cells in human pancreatic islets during the 72 h of culture. In the GS-Rd treated groups, the islets involved rare apoptotic cells, compared with those from the control groups. This reduction in TUNEL positive cells was not significant at 24 h (*p*=0.422). However, in all treated groups of 72 h incubation, apoptosis was significantly diminished (*p*<0.001) compared with the control group (Figure 5(Fig. 5)).

### Similar stimulation index of insulin and C-peptide between the studied groups

**Figure 6:**
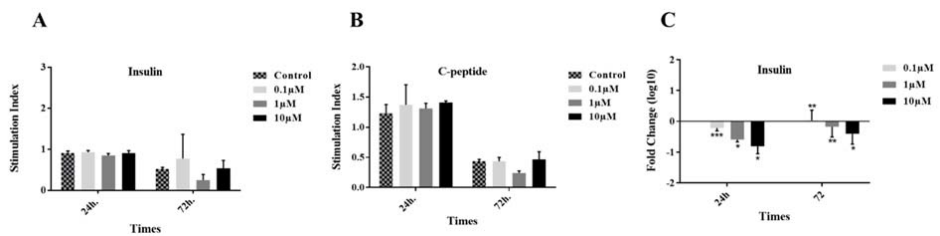
Insulin and C-peptide secretion assay. Insulin (A) and C-peptide (B) stimulation indexes were calculated by the response to 2.8 and 20 µM glucose. The insulin gene expression was also reported in three concentrations of GS-Rd at two time points (C). Results are mean ± standard error of mean (SEM).

After 24 and 72 h incubation of the islets in different groups, *in vitro* function was evaluated by measuring the stimulation indexes of glucose-stimulated insulin (Figure 6A(Fig. 6)) and C-peptide (Figure 6B(Fig. 6)) secretion. The differences between the results were not significant. Moreover, at the gene level, we found out that in the treated groups with 1 and 10 µM GS-Rd for 72 h and all concentrations for 24 h, the insulin was downregulated (Figure 6C(Fig. 6)).

## Discussion

**Figure 7:**
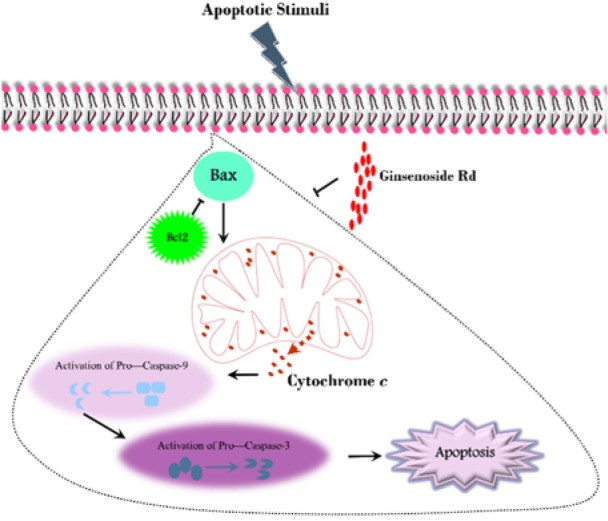
A schematic illustration of the intrinsic apoptotic pathway (in the area enclosed by a dotted line) and the effect of Ginsenoside Rd on this process

To date, islet transplantation has been suggested as an impressive method in the treatment of diabetes type 1. Although pre-transplantation culture provides an opportunity for analysis of the isolated islets, these cells deteriorate under this condition. According to the vulnerability of the pancreatic β-cells to oxidative stress and their low antioxidant content, recruitment of appropriate supplements can protect and recover the islets after isolation and during the culture. In the present study, the effect of GS-Rd was evaluated on the human pancreatic islets (Figure 7(Fig. 7)).

Many studies have evaluated the effect of GS-Rd on apoptosis and have yielded contradictory results. Many studies have revealed that GS-Rd remarkably promotes apoptosis in HeLa cancer cells (Yang et al., 2006[21]), basilar artery smooth muscle cells (Li et al., 2012[12]), and human glioma U251 cells (Gu et al., 2018[3]). On the other hand, increasing evidence shows that GS-Rd might behave as an anti-apoptotic molecule in some types of cells including the intestinal epithelial cells, cortical neurons, and hippocampal neurons (Li et al., 2010[13]; Liu et al., 2015[14]; Tamura et al., 2008[18]). Our findings are in line with those previous studies on the anti-apoptotic properties of GS-Rd. GS-Rd is known as one of the main components isolated from ginseng. Several studies have documented the safety and pharmacokinetics of GS-Rd. 

To the best of our knowledge, this is the first report on the impact of GS-Rd supplementation on the pancreatic islets apoptosis in the culture period. We found out that the inclusion of GS-Rd in culture media could suppress apoptosis of the pancreatic islets. In this regard, we analyzed the expression of BAX and BCL2 at gene and protein levels. We observed that the expression of BCL2 is upregulated, while BAX is downregulated in the presence of GS-Rd. In apoptosis, the intrinsic pathway is mediated by BCL2 family members. This family includes anti-apoptotic and pro-apoptotic members. The balance between these molecules, measured by *BAX/BCL2 *ratio, has an important role in the cell fate in the face of stressful conditions. Our results demonstrated that the presence of GS-Rd in human pancreatic islet culture media reduced this ratio. Immunohistochemically, we showed a substantial reduction in active caspase-3 in 10 µM and all studied concentrations of GS-Rd at 24 h and 72 h treatment, respectively. In line with the decrease of caspase-3 activation in treated pancreatic islets with GS-Rd, it significantly reduced the TUNEL positive cells. The evaluation of pancreatic islets viability with vital dyes revealed that the percentage of propidium iodide positive area in the untreated islets significantly increased after 72 h of incubation. However, GS-Rd maintained the cell viability during 72 h treatment.

Our findings are in accordance with those of Tamura et al.'s study that reported protective effects of GS-Rd against irradiation-induced apoptosis in the rat's intestinal epithelial cells (Tamura et al., 2008[18]). They showed that GS-Rd decreased the BAX/BCL2 ratio and the cleaved form of caspase-3. They also indicated that the studied cells were rescued from induced apoptosis through the activation of PI3K/Akt, inactivation of MEK, and also inhibition of mitochondrial-dependent pathway of apoptosis. Moreover, Liu et al. showed in their experimental study that GS-Rd attenuated apoptosis in primary cultured hippocampal neurons (Liu et al., 2015[14]). They found out that GS-Rd reverse Aβ25-35-induced oxidative stress and apoptosis alterations through the reduction of the activation of caspase-3 pathway. Li et al. also reported the preventive effect of GS-Rd on apoptosis in the cortical neurons. They indicated that this substance inhibited caspase-3 activation and the expression of p20 subunit of the cleaved caspase-3 (Li et al., 2010[13]). 

In the current study, we also evaluated the factors related to the function of pancreatic islets. Recent findings demonstrated that some active components of ginseng played a beneficial role in diabetes treatment. In our study, it was evident that the expression of insulin gene was reduced in a dose-dependent manner at 24 h and 72 h incubation in different concentrations of GS-Rd. Therefore, GS-Rd might have an adverse effect on the pancreatic islets function. On the other hand, GS-Rd did not present beneficial effects on the glucose induced insulin and C-peptide stimulation indexes. This finding is in accordance with that of Luo et al. that reported the lack of positive effects of GS-Rd on human islet β-cells insulin secretion (Luo et al., 2016[16]). They recently investigated the effect of ginsenoside Rb2, Re, Rg1, and Rd on human islet β-cells function. They concluded that only ginsenoside Rb2 increased the islet β-cells insulin secretion and others had no effect on insulin secretion *in vitro*. It has been proposed that Rb2 is likely to have a direct impact on the cells.

The mechanism underlying the effect of ginsenosides on glucose regulation is complex, with many contradictions. It has been indicated that ginsenosides can impress insulin production/secretion, glucose metabolism/uptake, and inflammatory pathway in diabetes. Previous studies have found that ginsenoside Rb1, Rg1, Rg3, and Rh2 possess insulin production and secretion activity in the studied cells (Yuan et al., 2012[23]). According to the mentioned beneficial effects of GS-Rd on the control of apoptosis in human pancreatic islets, it is suggested that this supplementation should develop with other concentrations of GS-Rd or combination with other molecules like ginsenoside Rb2 to improve the pancreatic islets function. Researchers reported that the effect of ginsenosides was different among human and animal studies. Therefore, the strength of our study, compared with the major studies that used animal pancreatic islets and pancreatic β-cell lines, is the recruitment of human pancreatic islets for the examination of apoptosis inhibition and islet function in the cell culture period before transplantation. On the other hand, an important criterion for the cell function is homing in their niche. So, it is suggested to implant the treated islets in animal models to determine the functionality.

## Conclusion

Overall, we attempted, for the first time, to evaluate the potential protective effects of GS-Rd on the inhibition of pancreatic islets loss during pre-transplantation culture. Our results demonstrated that GS-Rd may be a promising anti-apoptotic molecule for protecting the pancreatic islets. Moreover, these findings can provide a support for further studies about the effectiveness of GS-Rd.

## Acknowledgement

This study was a part of Maryam Kaviani's Ph.D. thesis (study ID: 94.521449). This project was financially supported by research deputy of Shiraz University of Medical Sciences. Authors are grateful to Dr. Nasrin Shokrpour for language editing of the manuscript and RCC Department of Shiraz University of Medical Sciences for statistical analysis consultation.

## Conflict of interest

The authors declare that they have no conflict of interest.
